# Promoting social and emotional competence in at-risk preschoolers: a mixed-methods evaluation of an SEL training program

**DOI:** 10.3389/fpubh.2026.1689775

**Published:** 2026-01-20

**Authors:** Keetam D. F. Alkahtani, Nouf Fadhel Althabeti

**Affiliations:** 1 Department of Special Education, College of Education, King Saud University, Riyadh, Saudi Arabia; 2 Taif University, Taif, Saudi Arabia

**Keywords:** Social and emotional learning (SEL), emotional and behavioral disorders (EBD), at-risk preschoolers, kindergarten intervention, positive behaviors support (PBS), teacher perspectives, teacher professional development

## Abstract

**Introduction:**

Preschool children at risk of emotional and behavioral disorders (EBD) frequently lack essential social and emotional learning (SEL) competencies. This mixed-methods study investigated the effectiveness of a targeted SEL program in addressing these developmental gaps and fostering positive behavioral outcomes in early childhood settings.

**Methods:**

The study employed a mixed-methods design involving 140 kindergarten children, including a sub-group of 29 preschoolers identified as being at risk for EBD. Quantitative data were gathered using a validated SEL assessment scale to measure pre- and post-program competence. Complementary qualitative insights were obtained through semi-structured interviews with six teachers who implemented the program.

**Results:**

Quantitative analysis revealed that the SEL program significantly increased competence levels among at-risk children, with statistically significant improvements across all measured domains regardless of gender. However, non-at-risk peers maintained higher overall competence levels throughout the study. Qualitative findings from teacher interviews mirrored these gains, noting improvements in social, language, and cognitive skills. Teachers also highlighted enhanced child-adult relationships and a reduction in negative classroom behaviors, while identifying specific environmental factors that influenced implementation success.

**Conclusion:**

Both data sets converge to confirm the efficacy of the SEL program for at-risk kindergarteners. The study concludes that such interventions are vital for fostering behavioral and social development, leading to recommendations for enhanced teacher training in SEL practices to foster positive behaviors in the classroom, improve classroom communication and student outcomes in early childhood settings.

## 1 Introduction

Social emotional learning (SEL) is now a core part of education, defined by the Collaborative for Academic, Social, and Emotional Learning (CASEL) as the skills needed to understand and manage emotions, set and achieve positive goals, build healthy relationships, and make responsible decisions (1). These abilities are vital for children’s development.

Many young children start school, typically kindergarten or first grade (ages 5–7), lacking sufficient social and emotional readiness, which increases their risk of emotional and behavioral challenges. This makes it crucial for educators to offer emotional support and teach conflict management. Social and Emotional Learning (SEL) programs, often implemented from preschool through elementary grades, can positively modify student behavior and improve the learning environment. Research (e.g., (2–7)) shows that these programs lead to better social–emotional skills, improved academic results, enhanced behavioral discipline, a more positive school climate, and better self-perception and perception of others among students. Children, particularly in the early elementary years, with strong social–emotional skills tend to behave better in the classroom and engage more effectively in learning (8–10). Conversely, those with weaker skills struggle to express or understand emotions, often leading to inappropriate reactions (8, 11–13).

Developing SEL during preschool years benefits children’s overall development and readiness for school. Children with strong social–emotional skills achieve higher academic performance, build more positive relationships, and show better emotional adjustment and mental health (12, 14, 15). The effectiveness of SEL programs is strongly supported by research highlighting their profound impact on young children’s development and school readiness. Studies emphasize their role in improving children’s ability to manage emotions, resolve conflicts, and interact effectively, contributing to well-rounded personalities capable of facing life’s challenges (16–18).

Specifically, developing SEL during preschool years yields significant benefits: children with stronger social–emotional skills often achieve higher academic performance, form more positive relationships, and exhibit better emotional adjustment and mental health (15, 19). Research suggesting that SEL interventions positively influence brain structure and function (20) supports the motivation to implement these programs in schools for preschoolers whose brains are undergoing rapid development. Systematic reviews and meta-analyses consistently confirm the effectiveness of classroom-based SEL in enhancing social and emotional competencies and reduce problematic behaviors, particularly for children needing early support (6, 21).

Early indicators of emotional and behavioral challenges in young children demand careful attention, particularly given that prevalence rates for significant difficulties in this domain may be as high as 20% among children aged one to seven, according to a meta-analysis by Vasileva et al. (22). While formal diagnoses of emotional and behavioral disorders (EBD) are typically applied to older age groups, these early difficulties profoundly affect children’s foundational academic readiness and social development. Children at risk of EBD often find it challenging to regulate their emotions and form reciprocal relationships with peers, which can hinder their successful navigation of early social environments. Developmentally, these struggles often manifest as negative self-perceptions, emergent low self-esteem, and social withdrawal, critically undermining their overall social well-being and setting the stage for later difficulties (23, 24). This underscores the urgent need for programs that support the mental and social well-being of children with or at risk for EBD. Implementing SEL programs is crucial as they empower these children to develop essential skills, resolve conflicts peacefully, and navigate life’s challenges more effectively.

This study addresses the critical need for holistic education that goes beyond academics to include social and emotional growth (25, 26). We will implement an in-school SEL program. The program’s primary goal is to improve social and emotional competence, helping children at risk for EBD learn to manage their emotions and interact more positively with others. Ultimately, this will give them the foundational skills they need to succeed both in and out of the classroom.

The purpose of the present mixed-method study was to investigate the effects of a SEL training program on the development of SEL competence in at risk preschoolers. We compared their performance before and after the program using a validated rating scale to identify changes in children’s SEL competencies. We also explored if the program’s effects varied by gender or the presence or risk of EBD. To gain a deeper understanding, we also gathered and analyzed teachers’ perceptions of the program’s impact on children’s social and emotional skills in the kindergarten environment.

## 2 Materials and methods

### 2.1 Participants

The study included two main participant groups for the quantitative and qualitative segments. The quantitative part of the study included 140 preschoolers aged 4–6 years (45.71% males, *n* = 64; 54.29% females, *n* = 76). Of these, 29 children (20.71%) classified as at risk for EBD if SDQ total difficulties fell within Borderline/Abnormal bands (teacher-report, Arabic version), while 111 (79.29%) did not. For the qualitative segment, teachers of these preschoolers participated. Their professional experience and relevant course history in EBD and SEL are detailed in Table 1. Ethical approval for the study was obtained prior to data collection.

**Table 1:** Participant profile: teacher experience and professional development courses.

Teacher	Years of Experience	Number of Courses	
		Related to EBD	Related to SEL
Teacher S	12	None	None
Teacher N	11	None	None
Teacher F	13	None	None
Teacher M	13	None	None
Teacher B	11	Two courses	None
Teacher J	11	One course	None

### 2.2 The social and emotional learning program

The SEL program implemented in this study is founded on the well-established content of “Zippy’s Friends” program. This particular program was selected for its documented effectiveness in enhancing social and emotional skills and mitigating problematic behaviors in students (16, 27, 28). It further aids students in developing adaptive coping mechanisms for daily life challenges and mental health difficulties (29, 30). Practical considerations also favored Zippy’s Friends due to its non-profit status, widespread international implementation, and availability in multiple languages (31). Zippy’s Friends is based on six stories about a group of three cartoon characters, their families, friends, and their pet stick insect named Zippy. Teachers read the stories and lead children through engaging activities like discussion, games, role-playing, and drawing to help them explore emotions, communication, and conflict resolution. Zippy’s Friends directly incorporates the five-core social and emotional learning competencies established by the CASEL (32), which serve as its foundational framework and guide the derivation of specific training procedures. Moreover, its design specifically addresses the characteristics of children with, or at risk for, emotional and behavioral disorders.

The program was delivered over a 10-week period, comprising four sessions per week, with a limit of one session per day within the classroom setting. Each session was approximately 45 min long, with flexibility for minor time adjustments as needed.

Implementation involved training teachers in the fundamental concepts of SEL and their practical application. To ensure robust implementation, fidelity was verified through an electronic guide and direct monitoring of teachers’ session delivery. Fidelity monitoring was conducted by two trained research assistants who used a 9-item checklist (see Appendix 1) to score adherence in a random 80% of all delivered SEL sessions. The rigorous monitoring procedures yielded high inter-rater reliability, with the monitors achieving 93% agreement. This strong consensus was confirmed by a high Cohen’s Kappa coefficient (*κ* = 0. 927). The electronic guide was a comprehensive digital resource provided to teachers, designed to outline the program’s specific lesson plans, instructional scripts, and required materials for each session. Social validity was assessed via interviews with participating teachers, who confirmed the program’s acceptance and ease of application within their classrooms.

### 2.3 Measures

#### 2.3.1 Strength and difficulties questionnaires (SDQ)

The Strength and Difficulties Questionnaire (SDQ), created by Goodman (33), serves as a behavioral screening assessment designed for children aged 2 to 17 years. Its utility is enhanced by its three versions—teacher, parent, and self-report (for 11–17-year-olds)—and its 25-item structure across five distinct sub-scales. These include four difficulty scales (Emotional Symptoms, Conduct Problems, Hyperactivity, and Peer Problems) and one strength-focused scale, the Pro-social Scale. Each sub-scale contains five items. Freely accessible online scoring keys facilitate the identification of children who may be at risk for behavioral and emotional disorders.

The SDQ was selected for this study due to its widespread use, ability to capture both difficulties and strengths, cost-free access, availability in numerous languages, and inclusion of a teacher report. Its straightforward administration, use, and scoring—typically taking only 5 to 10 min—further supported its selection for screening participating children and identifying those at risk. For this study, the official Arabic version of the SDQ was administered to participants via the teacher report. The Arabic version of the SDQ was accessed, and the scoring keys were utilized from the freely available resources at www.sdqinfo.org. For participant classification, we applied the standard banded cutoffs (Normal, Borderline, and Abnormal) to the total difficulties score. Children were subsequently classified as at risk if their total difficulties score fell within the Borderline or Abnormal ranges.

#### 2.3.2 The social and emotional competency rating scale (SECRS)

The Social–Emotional Competency Rating Scale (SECRS) developed by Brant and Studebaker (34, 35). They adapted the SECRS, a 42-statement instrument, based on two established assessments: The Devereux Early Childhood Assessment Rating Scale, DECA (36) and The Devereux Student Strengths Assessment, DESSA (37). This adaptation specifically involved content modification and selection to align the resulting 42 items with the five key social and emotional competency domains outlined by CASEL (1). The design of the SECRS focuses specifically on measuring these CASEL domains (as shown in Appendix 2). In the present study, the SECRS was utilized as a pre- and post-intervention measure, completed by the participating children’s teachers.

The internal consistency of the SECRS was assessed using two standard psychometric measures in the current sample. We calculated Pearson Correlation Coefficient between the scores of each SECRS subscale and the total scale score. The results demonstrated a high level of internal consistency, with correlations ranging from (*r* = 0.71) to (*r* = 0.83), as shown in Table 2. Furthermore, internal consistency was calculated. The overall scale displayed high constancy (*α* = 0.90). Similarly, the subscales demonstrated high constancy, with coefficients ranging from (α = 0.85) to (α = 0.92). The results are presented in Table 3.

**Table 2:** Pearson correlations between SECRS subscales and total score.

The SECRS subscale	Correlation coefficient (*r*)	Statistical significance
Self-awareness	0.71	<0.001
Self-regulation	0.79	<0.001
Relationship skills	0.76	<0.001
Social awareness	0.82	<0.001
Responsible decision-making	0.83	<0.001

**Table 3:** Internal consistency reliability of the SECRS subscales.

The SECRS subscale	Number of items	Cronbach’s alpha coefficient (α)
Self-awareness	7	0.92
Self-regulation	9	0.90
Relationship skills	9	0.91
Social awareness	9	0.89
Responsible decision-making	8	0.85
Total SECRS	42	0.90

#### 2.3.3 Semi-structured interviews

Semi-structured interviews were employed to gather qualitative data. This approach aimed to understand the program’s impact on participating children’s socio-emotional competence from the teachers’ perspective and to verify the accuracy of implementation procedures. Qualitative data was collected throughout and after the program’s execution. The interview protocol includes a total of eight questions, distributed across the program’s timeline. Participants are asked two questions during the pre-implementation phase (e.g., “From your perspective, what is social emotional learning?”), followed by three questions focused on their experience during implementation (e.g., “Describe your current experience applying the social emotional learning program in your classroom?”), and concluding with three questions in the post-implementation phase to evaluate their experience (e.g., “How do you evaluate your experience applying the social emotional learning program in your classroom?”).

Thematic analysis was used for data analysis, as it is a suitable method for qualitative data intended to strengthen, interpret, and better understand the quantitative data analysis results in this study. Thematic analysis helps explain phenomena by identifying recurring meanings or themes within the qualitative data. This process involved several stages, as outlined by Nowell et al., (38), which were followed in this study. The analysis utilized an inductive approach. Codes and themes emerged directly from the data, without a pre-existing theoretical framework. This ensured the findings were grounded in the teachers’ experiences. For objectivity, both authors independently coded all transcripts. They then met to compare, discuss, and reconcile initial coding discrepancies. This process led to a consensus on the final coding framework and its definitions. Reflexivity was maintained via research journals, which documented the rationale behind coding choices and thematic interpretations, mitigating researcher preconceptions and ensuring themes were rooted in the participant data. Specifically, pre-existing understandings of Social and Emotional Learning (SEL) were bracketed during the initial coding phase. This process yielded a high level of inter-coder reliability, achieving a 98% agreement with a strong Cohen’s Kappa coefficient (*κ* = 0.958). Credibility was established through member checking. A summary of the final themes and supporting quotes were presented to all teacher-participants, who verified that the analysis accurately represented their experiences and perspectives, thus validating the overall findings.

## 3 Results

The findings are presented in two parts: quantitative results from statistical analyses and qualitative insights from teachers’ perspectives.

### 3.1 Quantitative results

The effects of the intervention (Time: pre- vs. post-assessment), Sex, and Risk status on children’s Total Social–Emotional Competence were analyzed using hierarchical mixed-effects models. Children were nested within classes, and repeated measures were taken for each participant, reflecting the pre- and post-intervention assessments. This modeling approach allowed us to account for both between-subject variability (differences between children and classrooms) and within-subject correlations (changes over time).

Fixed effects included Time, Sex, Risk, and all possible two- and three-way interactions among these factors. Random effects consisted of random intercepts for children nested within classes, along with an unstructured covariance matrix for the repeated measures, permitting the estimation of variance and covariance between pre- and post-scores. Significance testing for fixed effects was conducted using Type III tests with Satterthwaite-adjusted degrees of freedom. Effect sizes for fixed effects were estimated via partial η^2^, providing a measure of the magnitude of each effect. For significant main effects, post-hoc pairwise comparisons with Bonferroni adjustment were performed to examine differences between levels of the factors. This comprehensive approach ensured that the analysis properly addressed the nested data structure, accounted for within-subject dependencies, and provided both inferential and descriptive information necessary for interpreting the impact of the intervention across different subgroups of children.

The mixed-effects analysis presented in Table 4 revealed a significant main effect of Time on Total Social–Emotional Competence, *F*(1, 136) = 253.96, *p* < 0.001, partial η^2^ = 0.651, indicating a large effect of the measurement occasion (pre vs. post).

**Table 4:** Type III tests of fixed effects for total SEL.

Source	Numerator df	Denominator df	*F*	*p*-value	Partial η^2^
Intercept	1.000	135.999	4735.708	<0.001	—
Sex	1.000	135.999	0.047	0.828	<0.001
Risk	1.000	135.999	39.073	<0.001	0.223
Time	1.000	135.999	253.962	<0.001	0.651
Sex * Risk	1.000	135.999	<0.001	0.986	<0.001
Sex * Time	1.000	135.999	0.491	0.485	0.004
Risk * Time	1.000	135.999	0.653	0.421	0.005
Sex * Risk * Time	1.000	135.999	1.075	0.302	0.008

The main effect of Risk was also significant, *F*(1, 136) = 39.07, p < 0.001, partial η^2^ = 0.223, suggesting that children’s risk status substantially influenced competence scores. No significant main effect of Sex was observed, *F*(1, 136) = 0.05, *p* = 0.828, partial η^2^ ≈ 0.

All two-way and three-way interactions between Sex, Risk, and Time were non-significant (all *p* > 0.05, partial η^2^ ≤ 0.008), indicating that the patterns of change across time were generally consistent across gender and risk groups. The variance component for the intercept (subject = ClassID * ChildID) was significant (Estimate = 0.0899, SE = 0.0253, Wald *Z* = 3.56, *p* < 0.001), indicating substantial variability between children nested within classes.

Mixed-effects modeling revealed significant pre–post improvements in total SEL scores. The intraclass correlation coefficient indicated modest classroom-level clustering (ICC = 0.13), suggesting that approximately 13% of the variance in total social–emotional competence was attributable to differences between classrooms, with the remaining variance occurring at the individual student level. Marginal *R*^2^ = 0.49 showed that fixed effects (Time, Risk, Sex) explained nearly half of the variance, while Conditional *R*^2^ = 0.56 reflected the contribution of both fixed and random effects. Residual plots indicated approximate normality and homoscedasticity, supporting the validity of model assumptions.

The unstructured covariance for the repeated measures (Time) showed a significant variance at Time 1 (Estimate = 0.1315, SE = 0.0338, Wald *Z* = 3.89, *p* < 0.001), whereas the covariance between Time 1 and Time 2 was not significant (Estimate = −0.0035, SE = 0.0224, Wald *Z* = −0.16, *p* = 0.875). The variance at Time 2 (UN 2,2) is reported as redundant due to model constraints.

These results presented in Table 5 support the appropriateness of a hierarchical mixed-effects model with random intercepts for children nested within classes and an unstructured covariance for repeated measures.

**Table 5:** Estimates of covariance parameters for total SEL.

Parameter	Estimate	Std. error	95% CI	Wald *Z*	*p*-value
Repeated measures UN (1,1)	0.131	0.034	[0.079, 0.218]	3.893	<0.001
Repeated measures UN (2,1)	−0.004	0.022	[−0.048, 0.040]	−0.157	0.875
Repeated measures UN (2,2)	0.119	—	—	—	—
Intercept [ClassID * ChildID]	0.090	0.025	[0.052, 0.156]	3.557	<0.001

Post-hoc pairwise comparisons presented in Table 6 indicate no statistically significant difference between males and females (*Δ* = −0.02, *p* = 0.828, Cohen’s *d* = −0.03, 95% CI [−0.21, 0.15]), indicating comparable levels of total SEL across sexes. Children classified as “not at risk” scored significantly higher than those “at risk” (Δ = −0.51, *p* < 0.001), with a moderate-to-large effect size (Cohen’s *d* = −0.60, 95% CI [−0.78, −0.42]), highlighting the impact of risk status on overall SEL. A large and statistically significant improvement was observed from pre- to post-intervention (*Δ* = 0.84, *p* < 0.001), with a very large effect size (Cohen’s *d* = 1.48, 95% CI [1.25, 1.71]), demonstrating the substantial effectiveness of the intervention.

**Table 6:** Pairwise comparisons of total SEL across sex, risk, and time.

Effect	Comparison	Δ	SE	95% CI (*Δ*)	df	*p*-value	Cohen’s *d*	95% CI (d)
Sex	Male – Female	−0.02	0.06	[−0.13, 0.09]	136	0.828	−0.03	[−0.21, 0.15]
Risk	At risk – Not at risk	−0.51	0.07	[−0.65, −0.37]	136	<0.001	−0.60	[−0.78, −0.42]
Time	Post – Pre	0.84	0.05	[0.74, 0.95]	136	<0.001	1.48	[1.25, 1.71]

**Figure 1:**
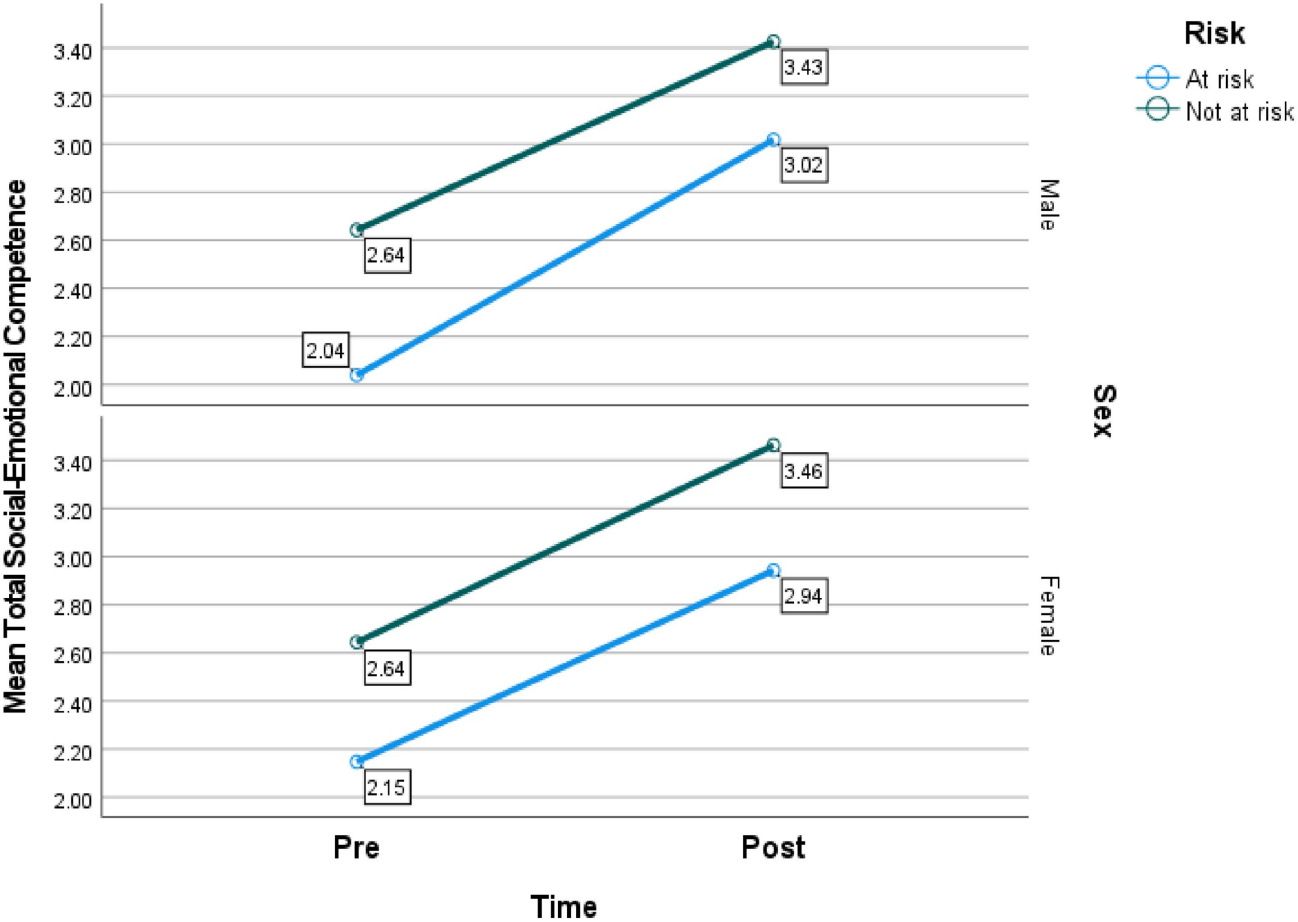
Mean total social–emotional competence scores by time (pre vs. post), sex (male vs. female), and risk status (at risk vs. not at risk).

Figure 1 presents mean total social–emotional competence scores. Among males, children at risk increased from 2.04 (pre) to 3.02 (post), representing a gain of 0.98, whereas children not at risk improved from 2.64 to 3.43, a gain of 0.79. For females, the at-risk group increased from 2.15 to 2.94 (+0.79), while the not at risk group rose from 2.64 to 3.46 (+0.82). Across all categories, post-intervention scores were higher than pre-intervention scores, indicating that the SEL program positively influenced children’s social–emotional competence. Although not-at-risk children consistently scored higher than at-risk children at both time points, both groups demonstrated substantial and relatively parallel improvements. Additionally, the patterns for boys and girls were highly comparable, suggesting no meaningful sex differences in response to the intervention.

Table 7 shows statistically significant pre–post improvements across all SECRS subscales as well as the total social–emotional competence score. Mean differences (Δ) ranged from 0.55 to 0.97, and all 95% confidence intervals were entirely above zero, indicating a consistent pattern of improvement across domains.

**Table 7:** Pre–post changes across SECRS subscales with Holm-adjusted *p*-values.

SECRS subscale	Δ (Post–Pre)	Std. error	95% CI (*Δ*)	*p*-value	*p* (Holm)	Cohen’s *d*	95% CI (d)
Self-awareness	0.965	0.075	[0.82, 1.11]	<0.001	<0.001	1.10	[0.88, 1.32]
Self-regulation	0.942	0.061	[0.82, 1.06]	<0.001	<0.001	1.32	[1.09, 1.55]
Relationship skills	0.552	0.072	[0.41, 0.69]	<0.001	<0.001	0.66	[0.48, 0.84]
Social awareness	0.884	0.068	[0.75, 1.02]	<0.001	<0.001	1.12	[0.90, 1.34]
Responsible decision-making	0.915	0.069	[0.78, 1.05]	<0.001	<0.001	1.14	[0.92, 1.36]
Total social–emotional competence	0.844	0.053	[0.74, 0.95]	<0.001	—	1.37	[1.13, 1.61]

After controlling for multiple testing using the Holm–Bonferroni procedure, all subscale effects remained statistically significant (*p* < 0.001), demonstrating the robustness of the findings and reducing the likelihood of Type I error inflation.

Regarding effect sizes, large effects were observed for most subscales, with Cohen’s d values ranging from 1.10 to 1.32. The Relationship Skills subscale showed a moderate-to-large effect (d = 0.66). The total social–emotional competence score exhibited a very large effect size (d = 1.37), indicating a substantial overall impact of the intervention. Confidence intervals for Cohen’s d were narrow and did not include zero, further supporting the stability and practical significance of these effects.

### 3.2 Qualitative results

Qualitative data were obtained from semi-structured interviews conducted with all participating teachers both during and after the implementation of the SEL program. The teachers’ responses provided their perspectives on the program’s influence on the children and the various factors that affected its implementation in the preschool setting. The data corpus consisted of 6 teacher interviews, with each interview lasting approximately 45 to 60 min.

**Figure 2:**
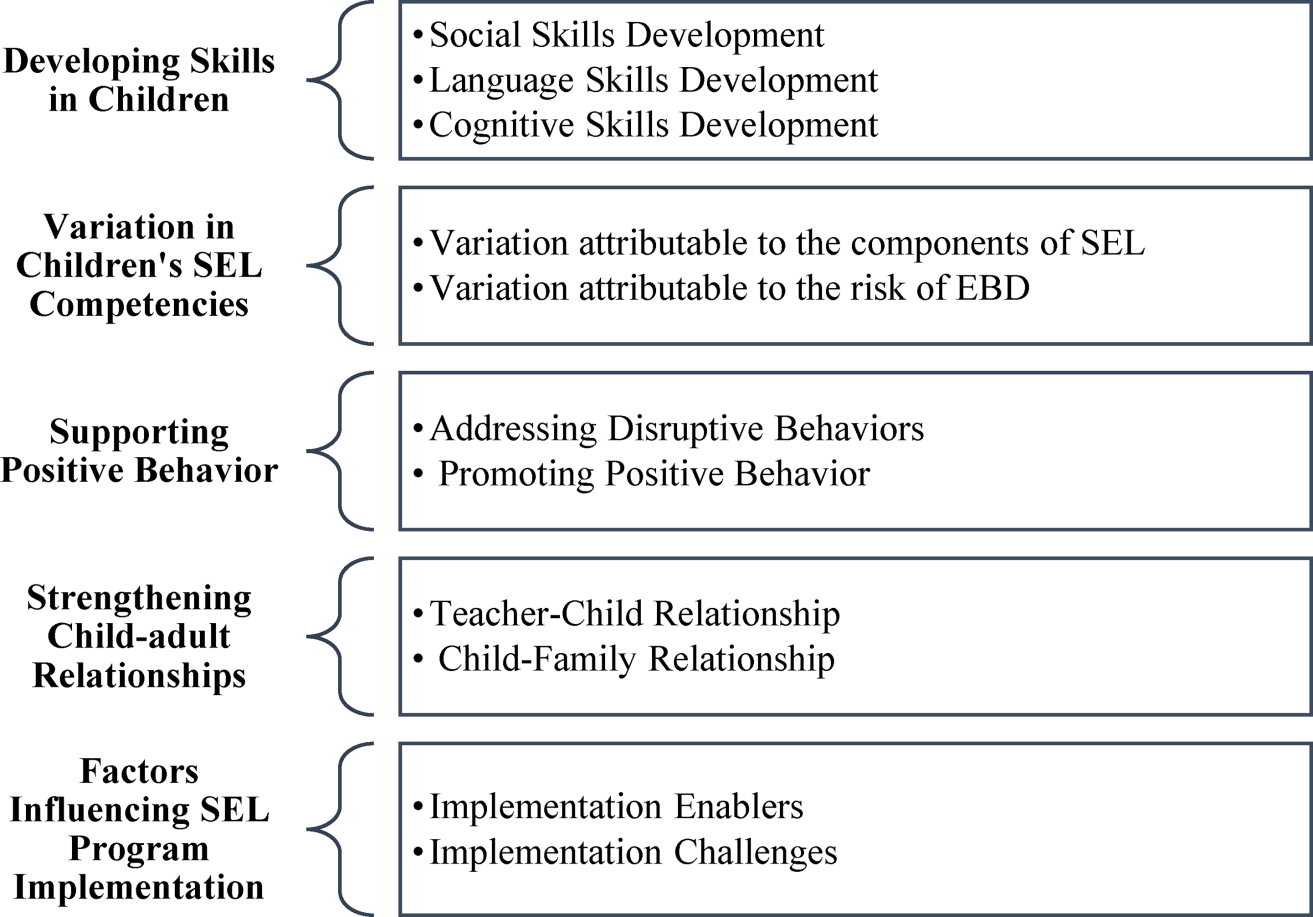
Thematic map: core themes and respective sub-themes identified through qualitative data analysis.

The collected narrative data were subjected to a thematic analysis (38) to uncover patterned meanings. This process involved six phases: familiarization with the data (transcribing and reading), generating initial codes, searching for themes, reviewing themes, defining and naming themes, and producing the final report. Five core themes emerged from the analysis: Developing Skills in Children, Variations in Children’s SEL Competencies, Supporting Positive Behavior, Strengthening Child-Adult Relationships, and Factors Influencing SEL Program Implementation. Figure 2 illustrates the themes and their corresponding sub-themes, and Table 8 provides illustrative quotes from the teachers.

**Table 8:** Participants’ supporting quotes on themes.

Quote numbers	Themes	Sub-themes	Illustrative quotes
Q1	Developing skills in children	Social skills development	Teacher S: The program really boosted the children’s social skills and their ability to communicate effectively. It did helped kids get better at getting along with each other and talking about what they think and feel. They started respecting how others felt and expressed themselves in healthier ways. I even saw massive improvements in the kids who struggled with emotional and behavioral issues and had a tough time with their feelings and behavior. They started being nicer to each other’s feelings, said what they needed to say in better ways, and even began caring more about their friends, like asking where a friend was if they were absent.
Q2		Language skills development	Teacher N: I observed remarkable growth in children’s linguistic expression and verbal skills, particularly their fluency of speech. At first, most of them just used one or two words to say how they felt. But now, they are using lots more words to talk about their feelings, even using new emotion words they learned from the program.
Q3		Cognitive skills development	Teacher F: I noticed that most children’s ability to pay attention to me and to what was happening around them improved, and the memory of most of the children got better. Kids with poor memory also started remembering the characters and important events in the story, and some of them even remembered the smallest details and events in sequence.
Q4	Variations in children’s SEL competencies	Variation attributable to the components of SEL	Teacher M: I think kids really benefited most from self-awareness unit. It really helped them in learning about themselves and get better at understanding and talking about their feelings. I also think that responsible decision-making was the area where I saw the least improvement. They did get a little better, but their age really plays a part here. They’re making decisions, but they are still too young to fully grasp the idea of being responsible for those choices.
Q5		Variation attributable to the risk of EBD	Teacher N: The program really boosted all the children’s abilities, which was great to see. Kids with behavioral and emotional challenges definitely benefited and grew too, though their progress wasn’t quite as significant as the others children.
Q6	Supporting positive behavior	Addressing Disruptive Behaviors	Teacher S: You know, I genuinely feel like this program had a big impact on the kids’ behavior, particularly for those who struggled with their behavior. Honestly, a lot of the bad behaviors that were pretty disruptive and annoying have stopped. For example, there was one child who used to pick on others almost every day, but after we went through the bullying part of the program, his behavior completely changed. I can tell you for sure that using this program helped fix those negative and disruptive behaviors, and because of it, the kids are just behaving much better overall.
Q7		Promoting positive behavior	Teacher B: I’m genuinely impressed by the swift and substantial improvements I’ve observed since implementing the program. There’s been a clear emergence of positive behaviors in almost all the children. For instance, I’ve witnessed them reminding each other of the learning rules in the corners—like saying, “No, what did we learn? We need quiet.” This has undoubtedly led to a significantly improved atmosphere across all learning environments.
Q8	Strengthening child-adult relationships	Teacher–child relationship	Teacher F: This program really opened things up between me and the kids. it allowed me to have really open chats with the kids, which helped me understand how they were feeling, what scared them, and what made them stressed. I also learned what they really enjoyed. Getting to know them so well made me feel closer to them, and they started to feel closer to me too. I think maybe it was because I understood them and their personalities so much better. I even noticed they started showing me more affection, and their behavior towards me got a lot better. I really believe that this positive connection we built made a big difference in how much the kids got involved in learning.
Q9		Child–family relationship	Teacher J: The relationship building unit created a ripple effect, improving relationships not only in the classroom but also within families. I saw significant improvements in family relationships. For instance, one mother shared that before the program, her daughter would just cry when she got angry, unable to express herself. Now, while she might still cry initially, she’s able to communicate what’s wrong when asked. This shift came from her understanding, post-program, that she needs to articulate her feelings so her family can help her. I also believe the program’s homework and exercises, which required parental involvement, were crucial to this success.
Q10	Factors influencing SEL program implementation	Implementation enablers	Teacher M: I think the biggest reason this program worked so well was because of its great stories and materials. The kids absolutely loved the stories—they were really good, and I think it’s because they were about things happening in their own lives. Plus, the questions and activities for each lesson were super clear and easy for us teachers to use. Honestly, at first, I was pretty nervous. I even thought about quitting! I kept thinking, ‘How am I going to teach something I’ve never learned about or had training in before?’ But once you gave us the training and showed us all the materials, and I saw in the workshop how much the kids would benefit, I got really excited and decided to stick with it.
Q11		Implementation challenges	Teacher B: Some kids missed a lot of class, and it really showed. The ones who came regularly got the most out of the program, no question. I also saw a huge difference when parents got involved. The kids whose parents talked to me and asked about the program really did the best. I honestly think if we had offered a workshop for parents to show them how to help, they would have gotten even more involved, and more kids would have done even better.

#### 3.2.1 Developing skills in children

Teachers consistently highlighted the significant positive impact of the SEL program on children. They observed improvements across three core developmental areas: social skills, language skills, and cognitive skills. All six teachers reported that the program significantly improved children’s social skills and communication, especially for at-risk students (Table 8, Q1). This supports previous research showing that SEL programs boost social skills (39) and increase social engagement in children with EBD (40). The researchers believe this is because social skills are a key component of SEL, particularly for preschoolers. During these early years, children start to understand how their actions affect social interactions, helping them take responsibility and manage their behavior (41). For children with EBD, early school interventions focusing on behavior and social aspects can significantly improve their often-low social and emotional performance (42).

All teachers reported an un-anticipated improvement in children’s language skills. They observed an increase in both spontaneous conversations and the use of social–emotional vocabulary during the program sessions (Table 8, Q2). While not a primary goal, the program led to an improvement in children’s language skills. This aligns with research from Taylor et al. (39) and a meta-analysis by Korpershoek et al. (43), both of which found that SEL programs positively impact students’ academic and language development. However, our findings diverge from Daunic et al. (44) study, which reported no such benefits on social–emotional vocabulary or language development. Daunic and colleagues suggested this might be due to the facilitators’ personalities and a lack of spontaneous conversations. Our study, in contrast, observed frequent and spontaneous interactions between children and teachers during our program sessions. This distinction is crucial because strong language skills are fundamental for SEL, empowering children to effectively express their feelings and interact with others.

Five out of six teachers reported that children participating in the SEL program developed stronger cognitive skills, including memory and attention. This improvement was especially noticeable in children prone to EBD (Table 8, Q3). This finding is consistent with a study by Voith et al. (45), which also noted improved concentration and attention in participating children in similar program. Researchers believe that this development of cognitive skills stems from an integrated approach to teaching social and emotional skills, one that focuses on all aspects of development, including cognitive ones (46). Furthermore, the program’s design in this study used a consistent set of characters across all six stories, with one story per unit. This consistent exposure likely reinforced children’s memory and attention skills.

#### 3.2.2 Variations in children’s SEL competencies

All six teachers observed varied individual progress in children’s SEL competence throughout the program. While all teachers noted improvement across every SEL component for all children, the most significant gains were consistently seen in self-regulation. In contrast, responsible decision-making showed the least improvement (Table 8, Q4). Five out of six teachers also noted that children at risk of EBD, while their progress was still considered significant, demonstrated less extensive improvement compared to their peers (Table 8, Q5).

#### 3.2.3 Supporting positive behavior

All six teachers observed that the SEL program successfully reduced undesirable behaviors (Table 8, Q6), a finding supported by various studies (16, 45, 47, 48) demonstrating SEL’s effectiveness in curbing bullying and other problematic conduct. This outcome is expected, as behavior modification is a core objective of SEL programs and aligns with their underlying cognitive theories (49).

Furthermore, all teachers noted that the SEL program also fostered positive behaviors, effectively acting as a preventive measure (Table 8, Q7). This aligns with research (6, 16, 30, 39, 50) indicating that SEL programs can prevent the development of future behavioral issues. This preventive impact likely stems from the program’s emphasis on teaching children expected behaviors through clear classroom rules and equipping them with the skills to meet these expectations—a strategy recognized as an effective early intervention for behavioral problems (51).

#### 3.2.4 Strengthening child–adult relationships

All six teachers reported a significant improvement in their relationships with children after implementing the program (Table 8, Q8). This positively impacted both the children and the teachers. This finding is consistent with existing research, including studies by Jaga et al. (52), Robinson-Ervin et al. (53), Özen et al. (54), Rucinski et al. (55), Sandilos et al. (56), and Voith et al. (45). These studies highlight that high-quality teacher-child relationships and a positive classroom emotional climate are crucial for children’s social, emotional, and academic development. Moreover, a study by Haymovitz et al. (23) noted that a key benefit of such programs is an improved classroom climate, leading to better teacher-child relationships and a safer school environment for children.

Five out of six teachers observed clear positive effects of the SEL program on participating children’s relationships with their families (Table 8, Q9). This observation is consistent with prior research, including studies by Grusec (57) and Li et al. (58), which highlighted the crucial role of family interactions in helping children develop social norms and interpersonal skills necessary for building strong, positive relationships.

#### 3.2.5 Factors influencing SEL program implementation

All six teachers reported that several factors influenced the SEL program, with some directly contributing to its successful implementation and goal achievement. Key among these was the program’s quality, particularly its reliance on engaging stories and interactive activities. The program also benefited from a comprehensive guide that detailed session objectives, procedures, and assessment methods. Furthermore, the training workshop provided to participating teachers was crucial for successful implementation (Table 8, Q10). These findings align with existing research. Voith et al. (45) similarly found that program quality and training impact the effectiveness of SEL programs for children. The importance of teacher training is also supported by studies from, Durlak et al., (2), Cipriano et al. (59), Hassani & Schwab (60), and Lawson et al. (61), which highlight training, along with the accessibility and clarity of educational content, as significant factors in SEL program implementation. Additionally, Haymovitz et al. (23) and Hutchins et al., (62) corroborate that training for program providers is vital for success, as it enhances the program’s positive impact on children.

Conversely, all six teachers identified factors that hindered the program’s maximum effectiveness. These challenges included children’s inconsistent daily attendance and a lack of active family participation (Table 8, Q11). This finding is consistent with research from Haymovitz et al. (23), Lawson et al. (61), and Li et al. (58) all of whom underscore the critical role of parental involvement in SEL programs, as family engagement provides a strong foundation for children’s social and emotional development.

## 4 Discussion

### 4.1 Efficacy of the SEL program

The quantitative results, showing significant improvement in overall SEL competence and a large effect size, align with previous research affirming the benefits of SEL interventions for at-risk children (27, 63, 64). However, these findings contradict some studies [e.g., (65)]. The discrepancies might be due to differences in sample age, data collection methods, and program implementation fidelity.

This program also significantly boosted all SEL competence, with self-regulation showing the most substantial improvement due to its effectiveness in helping manage behavioral issues. Self-awareness, social awareness, and responsible decision-making also saw significant gains. The improvement in responsible decision-making was particularly notable for kindergarteners, as the program’s emphasis on empathy helped them develop beyond typical egocentric behaviors. Finally, relationship skills improved significantly, likely as an indirect benefit of the advancements in other core SEL areas. The relationship skills showed a medium effect size, making it the least affected dimension. This might be because children at risk of EBD often exhibit disruptive behaviors like opposition and peer conflicts (62). Additionally, the development of relationship skills, given their social-dynamic nature, often requires extended learning periods (66).

These findings are consistent with existing literature [e.g., (6, 27, 30, 47, 53, 62, 64, 67)] affirming that SEL programs are effective in reducing negative behaviors and fostering greater responsiveness to adult guidance in students. The consistent improvements observed across all competencies, coupled with the substantial overall effect size, strongly support the SEL program’s effectiveness for this vulnerable population.

### 4.2 Influence of sex and disorder risk

The finding of no statistically significant difference based on sex in program effectiveness is supported by other research (29, 68). A likely explanation for this consistent improvement is that all children received the same structured program training, leading to a uniform positive effect.

Regarding risk of disorder, the finding that the program was more effective for children not at risk of disorders aligns with Sullivan et al. (69) who reported a stronger effect of SEL programs on non-disabled students. Conversely, it contrasts with Carroll et al. (68), who found the greatest impact on children identified as at risk of EBD. This discrepancy may be attributed to differences in the age groups studied—the current study focused on early childhood (kindergarteners), whereas Carroll et al. (68) examined children in late childhood. This developmental variation may account for the differing outcomes in SEL competence between children in these respective age ranges.

### 4.3 General discussion

This mixed-methods study aimed to comprehensively evaluate the SEL program’s impact, integrating results from both quantitative and qualitative analyses. The findings largely converged, offering rich insights into the program’s effects on children’s social and emotional competence, with some notable divergences that warrant discussion. Organizing the discussion by key SEL components and broader themes allows for a richer interpretation that moves beyond simple statistical findings.

Self-regulation showed the most significant improvement after program implementation, indicating children’s enhanced ability to manage their emotions and behaviors. Qualitative data strongly supported this finding, attributing the gains to the natural progression of behavioral development—a key objective of the program and a core principle of cognitive theories underlying SEL. The program’s focus on coping skills and stress management likely contributed to this substantial impact on self-regulation. The strong convergence of data confirms that the program was highly effective in enhancing children’s ability to manage emotions and behaviors. Teachers’ comments linked this to the program’s explicit focus on coping skills, which are particularly vital for children prone to EBD.

Responsible decision-making positioned as the second most impacted component while qualitative findings suggested it was the least extensive improved. This discrepancy might stem from the long-term developmental nature of this skill, which relies heavily on the advancement of self- and social awareness. Teachers’ qualitative observations may reflect a more complex, long-term perspective, suggesting that deep, responsible decision-making takes extended time and relies on the maturity of other SEL skills. The program’s success with kindergarteners on empathy-driven decision-making, however, is a positive indicator of foundational development.

Self-awareness and Social awareness showed significant improvement in the quantitative analysis. However, qualitative data indicated self-awareness as the most improved, possibly due to their focus on observable skills like identifying emotions. For social awareness, both qualitative and quantitative results aligned, confirming its enhanced role in fostering positive behavior.

Relationship skills appeared least affected by the program in the quantitative results, yet qualitative findings suggested considerable improvement. Quantitative data reflects smaller change from baseline, but qualitative observations capture the notable, daily relational dynamics that improved, possibly due to advancements in other core SEL areas. This divergence could be explained by children’s baseline skill levels before the program, as well as the deeper insights provided by qualitative data, which highlighted children’s daily relational dynamics.

Overall, the qualitative and quantitative results largely converged on the improvements across all SEL components. Furthermore, qualitative findings revealed the program’s broader impact beyond SEL competence, extending to linguistic and cognitive skills. The program also played a crucial role in promoting positive behavior, positioning it as a preventive intervention for EBD. The qualitative data also illuminated key success factors and challenges associated with implementing the program within school settings.

Quantitative analysis indicated the program was more effective for children not at-risk of EBD, a finding supported by teachers who observed less extensive gains in their at-risk students. This suggests that while the program is effective for both groups, children with existing behavioral risk factors may require a more intensive or extended intervention to achieve the same level of gain as their peers.

### 4.4 Broader benefits for the educational community

A significant finding from the qualitative analysis was the teachers reported a significant improvement in their relationships with the children and observed positive effects on the children’s family relationships. This suggests that the structured SEL curriculum not only benefits the children but also enhances the teaching environment and the quality of teacher-child interactions, which itself is a crucial factor in children’s development and reduces teacher stress (23, 52).

The success of the program was strongly linked to the quality of the program materials and the teacher training workshop. These findings affirm that engaging the educational community through professional development and providing accessible resources is an indispensable factor for effective SEL program implementation (2, 61). Conversely, challenges like lack of family participation underscore the need for future developments to more actively integrate the home environment to maximize a program’s potential.

## 5 Conclusion

The program significantly improved all SEL components, a conclusion strongly supported by both qualitative and quantitative results. Additionally, qualitative data highlighted the program’s wider influence, extending to enhancements in linguistic and cognitive skills. By promoting positive behavior, the program emerged as a crucial preventive intervention for EBD. Our qualitative analysis also shed light on the key factors contributing to the program’s success and the challenges faced during its implementation in school environments. Given these significant benefits, SEL program should be an indispensable element of the educational curriculum for kindergarten children, preparing them with the social and emotional competencies vital for navigating future challenges and achieving success.

## Data Availability

The dataset generated and analyzed for the current study contains human subject data. Therefore, to ensure participant privacy and adhere to the ethical approval granted for this study, data are not publicly available in an open repository. Access to the minimal dataset necessary to reproduce the findings can be requested from the corresponding author, subject to a controlled access agreement that includes confirmation of institutional ethics approval (IRB/REC) and a non-disclosure agreement. Data will be provided in a fully anonymized form, with all direct identifiers (e.g., names, dates, addresses) removed and indirect identifiers pseudonymized or aggregated to ensure the lowest risk of re-identification, consistent with the standards for human research data sharing.
