# Supplementary material for: Promoting social and emotional competence in at-risk preschoolers: a mixed-methods evaluation of an SEL training program
Source: Front Public Health. 2026 Jan 20;14:1689775. doi: 10.3389/fpubh.2026.1689775 (PMC12864509; doi:10.3389/fpubh.2026.1689775)
Supplement: Supplementary file 1 [file Data_Sheet_1.pdf]

frontiers

Appendix 1

SEL Implementation Fidelity Checklist

Observer: _________________ Teacher: _________________ Date: ___________

Rate on a scale of 1-3 (1 = Emerging, 2 = Proficient, 3 = Exemplary)
Scoring Rubric:
1: Emerging (Initial attempts; inconsistent or lacks depth, Minimal evidence).
2: Proficient (Consistent application; meets the standard, Clear evidence).
3: Exemplary (Integrated naturally; high student independence and mastery).

No.	Indicator	Rating (1-3)			Notes/Evidence & Observations
1	Physical Setup: Students are arranged (e.g., in a circle or on a rug) to facilitate eye contact and equitable engagement.	**1**	**2**	**3**	
2	Visual Supports: SEL tools (Mood Meters, feeling charts, "Calm Down" anchor charts) are visible and accessible.				
3	Explicit Naming: The teacher clearly identifies the specific SEL skill or objective (e.g., "Today we are learning how to 'stop and think'").				
4	Modeling: The teacher demonstrates the skill using a puppet, story, or personal modeling.				
5	Generalization: The teacher explains when to use the skill during the rest of the day.				
6	Emotional Validation: The teacher validates all feelings ("It's okay to feel frustrated") while redirecting unsafe behaviors.				
7	Common Language: The teacher uses program-specific vocabulary consistently to reinforce concepts.				
8	Active Participation: Students engage in role-play or collaborative work with high focus and minimal off-task behavior.				
9	Psychological Safety: Students feel safe enough to share a mistake or a difficult emotion without fear of judgment.				

Appendix 2

Social and Emotional Competency Rating Scale (SECRS)

Instructions: Please evaluate the child's behavior over the **past 4 weeks**. Select the frequency that best describes how often the child demonstrated each behavior using the following scale:

1 = Never **2** = Rarely **3** = Occasionally **4** = Frequently **5** = Very Frequently

CASEL's SEL Domains	SECRS's Items Description: During the past 4 weeks, how often did the child...	Rating (1–5)
Self-Awareness	Make accurate statements about events in her/his life?	
	Give an opinion on their own or when asked?	
	Ask questions to clarify what he/she did not understand?	
	Show an awareness of her/his personal strengths?	
	Seek affirmation?	
	Teach another person to do something?	
	Describe how he/she was feeling?	
Self - Regulation	Listen to or respect others?	
	Play well with others?	
	Cooperate with others?	
	Calm himself/herself down?	
	Share with other children?	
	Control his/her anger?	
	Show patience?	
	Handle frustration well?	
	Accept another choice when his/her first choice was not available?	
Relationship Skills	Has positive interactions with adults?	
	Ask adults to play with or read to him/her?	
	Trust familiar adults and believe what they say?	
	Seek help from children/adults when necessary?	
	Seem happy or excited to see his/her parents or guardian?	
	Shows affection for familiar adults?	
	Appear happy when playing with others?	
	Look forward to activities at home or school (for instance birthdays or trips)?	
	Shows a preference for a certain adult, teacher, or parent?	
Social-Awareness	Cope well with insults and mean comments?	
	Play well with different types of people?	
	Act respectfully in a game or competition?	
	Accept another peer's ideas?	
	Play well in a group?	
	Resolve a disagreement without teacher help?	
	Share with others?	
	Cooperate with peers?	
	Forgive somebody who hurt or upset her/him?	
Responsible Decision Making	Follow the example of positive role model?	
	Show the ability to decide between right and wrong?	
	Accept responsibility for what she/he did?	
	Learn from experience?	
	Seek help and use the advice of the teacher?	
	Use available resources (people or objects) to solve problem?	
	Show good judgment?	
	Ask for help?	
